# FACT-G Assessment of the Quality of Life for Palestinian Patients with Cancer

**DOI:** 10.5339/qmj.2022.43

**Published:** 2022-09-01

**Authors:** Carol El Jabari, Inad Nawajah, Hussein Jabareen

**Affiliations:** ^1^College of Nursing, Hebron University- Palestine inadn@hebron.edu; ^2^College of Science and Technology, Hebron University- Palestine

**Keywords:** QOL, Palestine, patients with cancer, palliative care, FACT-G, Statistics, ANOVA

## Abstract

Background: Cancer is a leading cause of mortality in Palestine. The number of cancer cases is increasing, whereas the late stage of diagnosis is common for the majority of cases. Modern diagnostics and medicine are contributing to more positive outcomes for patients when diagnosed early; however, the holistic approach to patient care, patient satisfaction, quality of life (QOL), and survivorship are often overlooked. Patients with cancer are usually treated by physicians and other health professionals employing the “medical model” without considering other factors that might positively affect their treatment. For this pioneering survey, the Functional Assessment of Chronic Illness Therapy-General (FACT-G) tool was used. This study aimed to measure the satisfaction of patients with cancer undergoing outpatient treatments and assess their QOL.

Materials and Methods: The FACT-G scale in Arabic has four sections, measuring physical, emotional, social, and functional well-being, was used in a survey of 203 patients with cancer currently undergoing chemotherapy, radiotherapy, and hormonal therapies. Patients were surveyed in the summer of 2019, within 1 year of diagnosis when they were outpatients, all of whom were attending a major cancer center in a Palestinian hospital.

Results: Emotional well-being scored the lowest (from a total of 24; mean 11.31 (standard deviation (SD) 5.45)) whereas social/family well-being scored the highest (from a total of 28), mean 22, (SD ±  5.78). The overall mean for the four sections was 63.57 (SD 12.44).

Conclusion: To ensure better management of symptoms and treatments, important indicators are now available for health professionals and researchers to learn more about the QOL of patients. Evaluating the physical, social, emotional, and functional states of patients with cancer undergoing outpatient treatments enabled us to learn more about the struggles they face while understanding how they were coping during their cancer journey.

## Introduction

The cancer burden is increasing in Palestine. According to the Palestinian Ministry of Health, the cancer incidence rate was 117.8/100,000 populations in 2019. There were 3,174 new cancer cases in the West Bank, with breast cancer being the most common cancer (n = 536, 16.9%), with an incidence rate of 19.9 per 100,000 populations. This was followed by colon cancer (12.6%), lung cancer (7.2%), thyroid cancer (5.2%), leukemia (4.9%), and bladder cancer (4.7%).^1^

Worldwide, cancer is a leading cause of death, accounting for nearly 10 million deaths in 2020.^2^ Mortality rates were 41.9 and 36 deaths per 100,000 people in the West Bank and Gaza, respectively.^3^ Data from the Gaza Strip is not available. According to Al Waheidi in Gaza, “a lack of complete and reliable data remains a major challenge.”^4^

Late diagnosis of cancer is a persistent problem in the country, with at least 60% of cases being diagnosed at stage 3 or 4, which often results in more aggressive treatments and poorer outcomes. The World Health Organization (WHO) projections for the Eastern Mediterranean region state that 630,000 cancer deaths will occur in 2030, an increase of nearly 57% from 2015.^3^

Quality of life (QOL) is important in cancer care because it influences both the disease and treatment. As effective methods of treatment and detection have led to an increase in the number of long-term survivors, healthcare providers should understand the factors that may contribute to the QOL of their patients.^5^

This study mainly aimed to assess the health-related (HR)-QOL of Palestinian patients with cancer by using a validated questionnaire. Patients who participated in this survey came from all backgrounds and from all parts of the country.

QOL is a multi-dimensional construct that affects all aspects of a person’s life including physical health, mental and social well-being, and functional factors. It is also influenced by cultural, ethical, personal, and religious values.^6^ Arab women share different cultures, norms, and beliefs compared with Western women, and studies have shown that patient-based outcomes could be affected by cultural experiences and ethnic backgrounds.^7^

Islam is the dominant religion in Palestine, 98% are Sunni Muslims, with a population of >4.8 million in the West Bank and Gaza.^8^ Many Muslims believe that death and illness are the will of Allah (God) and cannot be avoided or stopped.^3^ Abu Huraira reported Allah’s Messenger, peace be upon him, saying: “For every misfortune, illness, anxiety, grief, or harm that afflicts a Muslim -even the harm is caused by the pricking of a thorn - Allah removes some of his sins.” They see illness as a test from God. While religion encourages seeking advice and treatment, Islam also teaches that tolerating pain without medications or treatments will lead to greater rewards in the hereafter. This belief helps the patients to cope.

Family support for patients is another factor that affects QOL.^9^ The Palestinian society and family structure is extended, with frequent family gatherings, children are living at home until they are married. The decision-making for families usually involves numerous relatives. Christian and Muslim families have similar values and traditions, such as respect for the elderly and care for the weak and ill.^3^

In Palestine, limited access to care and financial constraints play an important role in the QOL of patients with cancer. The health sector suffers economically and has a weak infrastructure. The Palestinian Authority (government) provides health cover for patients with cancer, but the overall health expenditures are insufficient to meet demands. The total health expenditure per capita in Palestine (excluding east Jerusalem) was USD 282.2 in 2015, whereas the Palestinian Central Bureau of Statistics reported that at least 40% of costs must be paid by the patients and their families. Palliative care is not a priority and is not covered by governmental health insurance, which covers the majority of the population. However, the non-governmental sector attempts to educate health professionals, improve basic services, and expand diagnostic and psychosocial support. The first support group for women living with cancer began in 2000, which was established by Patient’s Friends Society-Jerusalem, a non-governmental organization. Their work encompasses psychosocial support, lymphedema care, education, advice, etc., for women and their families from the West Bank and Gaza. Health professionals received some training in oncology and palliative care, but such trainings are donor dependent and services are sporadic.

The West Bank has two governmental hospitals and two non-governmental hospitals that provide curative and palliative care treatments, whereas Gaza has three hospitals. Radiotherapy is not available in the West Bank or Gaza. As the referrals of Palestinian patients are approved and they obtain travel permits from the Israeli occupation forces, they may access radiotherapy at Augusta Victoria Hospital, Jerusalem.

Palliative care and a specialized focus on HR-QOL are limited and are in their infancy in Palestine. Moreover, very little data and research about HR-QOL for patients with cancer are available in Palestine, which may indicate the lack of concern, awareness, or interest in this field.

## Methods

This study utilized the Functional Assessment of Cancer Therapy – General (FACT-G) questionnaire (Part 2 of the research), which has 27 items designed to measure four domains of the HR-QOL in patients with cancer: physical, social, emotional, and functional well-being. The FACT-G is one of the most widely used patient reported outcome measures in cancer research.^10^ This questionnaire is designed for self-assessment.

The FACT-G survey scores physical well-being (PWB; 7 items, score range 0–28), social/family well-being (SWB; 7 items, score range 0–28), emotional well-being (EWB; 6 items, score range 0–24), and functional well-being (FWB; 7 items, score range 0–28).

All questions in the FACT-G use a 5-point rating scale (0 = not at all; 1 = a little bit; 2 = somewhat; 3 = quite a bit; and 4 = very much). Provided that >50% of the items comprising a subscale are answered, a subscale score is computed as the pro-rated sum of the item responses for that subscale. The FACT-G total score is computed as the sum of the four subscale scores provided that the overall item response is at least 80% (i.e., at least 22 of the 27 items were answered) and has a possible range of 0–108 points.

In addition to using this tool, a section (Part 1) was developed to survey demographic characteristics. These variables included age, sex, education, and monthly family income. Questions about metastasis and comorbid conditions, developed by the authors, were also included. Patients reported if they had metastasis or any chronic medical conditions. Recruitment and data collection were completed in 1 month during July 2019.

### Sample

The study enrolled adult patients with cancer who had been diagnosed at least 1 year previously and were attending a major referral hospital in Jerusalem. Patients with cancer came from all over the country and were receiving cancer treatment and/or follow up in the outpatient units (n = 203). A total of 157 (77.3%) patients were recruited from the West Bank and 46 (22.7%) from the Gaza Strip. The response rate was 98.5%.

All the patients were diagnosed with solid tumors (age range, 18–65 years; male, n = 65; female, n = 138 (Table 1). The patient reported comorbidities at the time of the survey (n = 88, 43%). The most common conditions reported were hypertension and diabetes.

### Data

The Functional Assessment of Chronic Illness Therapy system of QOL questionnaires and all related subscales, translations, and adaptations were developed by David Cella. The questionnaires were prepared, organized, and classified with serial numbers to ensure the availability of the needed information. The self-administered, two-part questionnaires in Arabic were distributed to the convenience sample of volunteers and returned immediately. The researcher explained the purpose of the questionnaire to the participants before obtaining consent and during the encounter and clarified any unclear information. Trained data collectors provided assistance when necessary or when the patients requested to complete the questionnaires. The average time for questionnaire completion was 15 min. Patients were asked to report on their QOL for the last 7 days before answering the questions. The study was explained to the participants, and verbal consent was obtained from each of them. The study was approved by the Hebron University Ethics committee.

## Results

A total of 203 adult patients with cancer responded to the questionnaires. The first section of the instrument included personal characteristics and demographic data, and the second section included the validated QOL questionnaire (FACT-G).

All FACT-G scales were scored so that a higher score indicated a better QOL. To achieve this, we reversed response scores on negatively phrased questions and then summed item responses. The negatively phrased questions that were reverse coded were in the subscales. In cases where individual questions were skipped, scores were pro-rated using the average of the other answers in the scale. The total FACT-G score was obtained by summing individual subscale scores (PWB+EWB+SWB+FWB). Total scores for the disease-, treatment-, and condition-specific subscales were obtained by summing all subscale scores (PWB+EWB+SWB+FWB).

Means and standard deviations (SD) were used to describe all domains (PWB, SWB, emotional EWB, and FWB). Data were tabulated using the IBM SPSS Statistics, version 22.0 (IBM Corp., Armonk, NY, USA),^11^ and possible associations between QOL and the following variables were analyzed: sex, age, education level, marital status, type of cancer, time since diagnosis.

The inter-reliability coefficient of FACT-G was indicated by Cronbach’s alpha 0.91, indicating high internal consistency. This result meets the criteria that Hahn et al. discussed in their article on “Precision of health-related quality of life data compared with other clinical measures.” They stated that “common thresholds for acceptable reliability are >0.70 for group-level applications and >0.90 for individual level applications.”^12^

Patient characteristics are demonstrated in Table 1. The majority of the participants were women (68%). Moreover, 75% of all participants were married. Approximately 40% of the participants were illiterate or had only elementary education, whereas nearly 16% had a university degree. In addition, 48.3% of the participants were between 40 and 59 years old, and >10% of the participants reported living in refugee camps, 53% in cities, and 36% in villages.

The mean* ± *SD total scores of the PWB, SWB, EWB, and FWB subscales of FACT-G are depicted in the following table. The maximum possible score is 108.

As shown in Table 2, EWB had the lowest mean (11.31), whereas SWB had the highest mean (22). The mean FACT-G total score in Gaza was higher (66.87 (SD 11.77)) than that of West Bank (62.61 (SD 12.48) (t-test = 3.44, *p* = 0.001)). The total mean and standard deviation scores of the items of PWB, SWB, EWB, and FWB subscales of the FACT-G are presented in Table 3.

In this study, the most common cancers included breast, colon, stomach, and chronic myeloid leukemia (CML). Twenty-six patients reported metastatic cancer. In Table 4, we focused on these cancers to demonstrate patients’ self-reporting. The overall FACT-G score for women with breast cancer was 64.52 (SD 13.73), whereas patients with CML scored 60.42 (SD 14.19). The p-value of 0.59 (chi-square = 14.56) indicated no significant difference.

No significant difference was found when QOL was compared between men and women (t-test =  2.34, *p* = 0.16). Women scored slightly higher in the PWB, FWB, and SWB subscales (Table 5).

## Discussion

In general, research is limited in Palestine regarding the QOL of patients with cancer.^13^ To our knowledge, this is the first of such survey conducted in Palestine using the FACT-G Arabic version. However, this study is limited by the small sample size, albeit representative. More than 40% of the patients reported comorbidities, especially among those aged ≥ 40. Thus, it is difficult to conclude that these patients were able to report or relate their QOL solely on their “cancer condition.” Bottomley^14^ mentioned that some researchers find an aggregation of scores helpful to assess QOL measures while discussing the possibility of the patients to focus more on limited physical functioning, as their condition worsens.

SWB scored the highest, with no significant difference between sex, scoring a mean of 22 (SD 5.75) of a total of 28. These are similar to the findings of Conrad et al.^15^ and Nayak et al.^16^ who discuss that this may reflect the greater importance of these domains for these patients. Women scored higher than men in FWB (mean 15.99/SD 6.15 of 28), whereas men scored slightly higher for PWB (15.88/SD 6.02 in men vs. 14.72/SD 7.07 in women). These differences were not significant.

EWB scored the lowest, with “I am losing hope in the fight against my illnesses” and “I worry about dying” being the worst among male and female respondents equally (mean 11.30/SD 5.40 of a total of 24). Similarly, in their study of Indian patients, Nayak et al. found that the fear of recurrence (76%) had the most effect on EWB.^16^

Evaluating the QOL of patients with cancer is increasingly important.^17^ QOL depends on many variables, such as access to information, sound physician–patient communications, and participation in the decision-making process including treatment options.^18^ Studies have shown that high levels of support, regardless of source, improve the ability to cope. Rockoff et al.^18^ analyzed 100 patients with breast cancer and survivors and found that better interaction and communications with health professionals (30%) and access to information (25%) could improve the treatment process and QOL. Coping was made more difficult by the clinical process (25%), perceived lack of control (17%), and fear of cancer and death (20%).

Patient data results may vary among populations and patients and should be considered in assessing patients and planning for their care.^14^ Non-communicable diseases (NCDs) in patients with cancer are associated with a poorer QOL and higher mortality than in patients without NCDs. According to the WHO, NCDs are the leading cause of death and a major economic and social burden in Palestine. For example, in the Palestinian population in the West Bank, Gaza, and east Jerusalem, there is a high prevalence of diabetes, with 15.3% compared with the worldwide prevalence of 6%. The World Diabetes Foundation further reports that anecdotal information from several sources suggested that the rate could be much higher, 18%–21%. Of the total diabetic population in Palestine, 4.4% and 95.3% are diagnosed with type 1 and 2 diabetes, respectively.

Clearly, 43% of the patients reported comorbidities, which were NCDs. Moreover, 16% of our patients reported having diabetes, whereas 21.5% reported having hypertension. Roy et al.^19^ stated that “increase in comorbidities can have significant implications for cancer patients.” Therefore, it is important to consider comorbid conditions when studying quality of care and survivorship.^20^ In the present study, all patients were currently receiving various treatments; some traveled long to receive care, and at least 13% were living with metastatic disease. None reported secondary cancers, but this was only ascertained from their self-reporting. While patients have the right to know their diagnosis, many reported not being told about the extent of their disease or prognosis.

The most highly-scored section was the SWB (22/28 score). The family is very important in the Palestinian culture, and generally, they offer as much support as possible. Similar experiences were found by Turkish and Moroccan patients in Europe.^21^ They also stated that culture affects communication, decision-making, response to symptoms, treatment choices, and emotional expression at the end of life. Faith and spirituality are the keys to culture, which provide patients enormous comfort and solace. In Islam, of which the majority of our samples are adherents, all life aspects are viewed within the context of religion. These findings are similar to those of Rockoff et al.^18^ In that study, women reported that the most difficult stage of cancer experience was its diagnosis. We might conclude that the study participants had moved on from this difficult stage and were adapting or coping better.

Generally, women scored slightly higher on the FACT-G (mean 64.20, SD 12.98) than men (mean 62.25, SD 11.12). Young men (aged < 18 years) and older men (60 years) tended to score less, whereas women scored low in the PWB, which may be because the majority of female respondents had breast surgeries affecting self-image, etc.

A patient with cancer stated: “Quality of life is so important after cancer, but many of us have a very poor QOL and are expected to just be grateful that we are still alive. It is so hard when everything you had before cancer is gone. Just being alive is not enough for me.”

Islam MS points out that “with the improvement of therapies patients with malignancies are living longer but the physical, social and psychological impacts on them often negatively impacts their QOL. Surviving cancer is a chronic life-altering condition’ which needs a holistic approach to patient care.”^22^

## Conclusions

To our knowledge, this was the first survey conducted in Palestine using the FACT-G Arabic version. This study indicated that patients with cancer experience symptoms such as nausea, feeling ill, and other problems, mostly emotional, that affect their QOL. As health professionals, it is incumbent on us to work together to ensure that patients receive timely care and support and thus improve their QOL. The main issues are symptom management and the need to use strategies that will empower patients to have a better sense of control over their illness and treatment. If we are to improve care, healthcare providers, including the government which is the largest healthcare provider in the country, and the academia should invest and commit to improving services; educate physicians, nurses, and other healthcare service personnel; support research; and implement strategies to help patients with cancer and their families.

We believe that this study will encourage other professionals to continue to, explore, and assess meaningful data and factors that affect patients and design interventions to improve QOL. This should be the future to ensure quality cancer services.

“The author(s) declare(s) that there is no conflict of interest regarding the publication of this article.”

We would like to acknowledge the contribution of FACIT.org who provided the tools and advice.

## Figures and Tables

**Table 1:** Participant characteristics (n = 203)

Variable	Description	N	%

Sex	Male	65	32

	Female	138	68

Age	≤ 20years	7	3.4

	21–39 years	43	21.2

	40–59 years	98	48.3

	≥ 60 years	55	27.1

Marital status	Married	155	76.4

	Single	29	14.3

	Widow/widower; divorced	19	9.4

Education	None	23	11.3

	Primary (up to 6th grade)	57	28.1

	Secondary	67	33

	Diploma	24	11.8

	Undergraduate and higher	32	15.8

Household financial status/month in shekels^*^	< 15001500–3000 ≥ 3000	667760	32.537.929.6

Employment status	Currently employed	30	14.87

	Retired	142	70

	Housewife	31	15.2

Residence	Village	72	35.5

	City	108	53.2

	Camp	23	11.3


^*^Note: 1 USD = 3.60 shekels

**Table 2:** Summary of FACT-G scales and subscales (mean and standard deviation for Palestinian patients with cancer), n = 203

Scale	Number of items	Score range	Mean (SD)

Physical well-being	7	0–28	15.08 (6.76)

Social/family well-being	7	0–28	22.00 (5.78)

Emotional well-being	6	0–24	11.31 (5.45)

Functional well-being	7	0–28	15.19 (6.31)

FACT-G total score	27	0–108	63.57 (12.44)


**Table 3:** FACT-G mean and standard deviation (SD) of each subscale item, n = 203

Subscale item	Mean	SD	Range

PHYSICAL WELL-BEING			

I lack energy	2.06	1.25	0–4

I have nausea	1.79	1.37	0–4

Because of my physical condition, I have trouble meeting the needs of my family	2.47	1.27	0–4

I have pain	2.45	1.24	0–4

I am bothered by the side effects of treatment	2.34	1.35	0–4

I feel ill	2.14	1.40	0–4

I am forced to spend time in bed	1.83	1.43	0–4

Total	15.08	6.76	0–28

SOCIAL/FAMILY WELL-BEING			

I feel close to my friends	2.87	1.22	0–4

I get emotional support from my family	3.47	0.84	0–4

I get support from my friends	3.06	1.20	0–4

My family has accepted my illness	3.43	0.96	0–4

I am satisfied with family communication about my illness	3.36	1.02	0–4

I feel close to my partner (or the person who is my main support)	3.23	1.20	0–4

I am satisfied with my sex life	2.61	1.76	0–4

Total	22	5.78	0–28

EMOTIONAL WELL-BEING			

I feel sad	1.99	1.27	0–4

I am satisfied with how I am coping with my illness	2.68	1.32	0–4

I am losing hope in the fight against my illness	1.49	1.46	0–4

I feel nervous	2.03	1.40	0–4

I worry about dying	1.20	1.42	0–4

I worry that my condition will get worse	1.91	1.53	0–4

Total	11.31	5.45	0–24

FUNCTIONAL WELL-BEING			

I am able to work (include work at home)	1.68	1.27	0–4

My work (include work at home) is fulfilling	1.74	1.27	0–4

I am able to enjoy life	2.03	1.33	0–4

I have accepted my illness	3.02	1.11	0–4

I am sleeping well	2.12	1.27	0–4

I am enjoying the things I usually do for fun	2.11	1.30	0–4

I am content with the quality of my life right now	2.52	1.31	0–4

Total	15.19	6.31	0–28


**Table 4:** Mean and standard deviation of the subscales grouped by the most common types of cancers reported by patients in this study

	Physical well-being		Social/Family well-being		Emotional well-being		Functional well-being		FACT-G Total score	

Cancer type	Mean	SD	Mean	SD	Mean	SD	Mean	SD	Mean	SD

Breast n = 75	15.27	6.77	21.77	6.25	12.12	5.63	15.36	6.21	64.52	13.73

Colon n = 30	15.87	7.07	21.33	6.44	11.90	5.13	13.83	7.50	62.93	14.95

Metastases n = 26	15.23	6.84	22.11	4.97	10.65	5.95	14.77	5.68	62.77	9.27

Stomach n = 14	13.85	6.47	21.50	5.54	10.86	4.53	14.86	5.55	61.07	11.20

CML n = 12	14.08	8.19	21.83	7.95	9.16	6.30	15.33	5.82	60.42	14.19


CML, chronic myeloid leukemia

**Table 5:** Summary of FACT-G scales (mean and standard deviation) for Palestinian patients with cancer grouped by age and sex

		Female				Male			

n		10	52	76	All	9	16	40	All

Age		< 29	30–49	≥ 50	138	< 29	30–49	≥ 50	65

PWB	Mean	11.8	16.4	13.9	14.1	12.4	17.3	16.1	13.4

	SD	4.3	3.2	3.4	3.8	7.01	6.9	4.2	5.1

SWB	Mean	25.2	21.5	22.3	23.1	23.3	20.4	21.6	22

	SD	2.6	3.3	4.6	3.6	5.2	7.5	3.2	4.6

EWB	mean	9.1	11.6	11.4	10.2	11.6	14.2	10.5	12.3

	SD	3.4	3.4	3.6	3.3	5.8	3.2	4.1	4.2

FWB	Mean	15.7	16.1	15.9	15.6	14.6	11.9	13.9	12.6

	SD	3.4	4.2	3.6	3.8	5.2	4.3	4.2	4.6

Total	Mean	61.8	65.6	64.6	63.2	61.9	63.8	61.7	62.3

	SD	4.2	3.9	3.4	3.8	4.2	3.4	4.2	3.9


PWB, physical well-being; SWB, social well-being; EWB, emotional well-being; FWB, functional well-being
